# Biological Screening of Euphorbia *Aellenii*

**Published:** 2010

**Authors:** Abdul Majid Ayatollahi, Mustafa Ghanadian, Suleiman Afsharypuor, Sadia Siddiq, Seyed Muhammad Pour-Hosseini

**Affiliations:** a *Phytochemistry Research Center and School of Pharmacy, ShaheedBeheshti University of Medical Sciences, Tehran, Iran*.; b *Isfahan Pharmaceutical Sciences Research Cente*r*, Isfahan University of Medical Sciences, Isfahan, Iran.*; c *Dr.Panjwani Center for Molcular Medicine and Drug Research, International Center for Chemical and Biological Sciences, University of Karachi,Karachi, Pakistan.*; d *Medical Genetic Department, Faculty of Medicine, ShaheedBeheshti University of Medical Sciences, Tehran, Iran.*

**Keywords:** *Euphorbia Aellenii*, Phytotoxicity activity, Insecticidal activity, Anti-leishmanial activity, Cytotoxicity activity, Antibacterial activity, Antifungal activity

## Abstract

Fractions obtained from partitioning of aerial methanolic extract of *Euphorbia Aellenii*with chloroform (E2), ethyl acetate (E3), *n*-butanol (E4) and water (E5) were investigated *in vitro *for their phytotoxicity, insecticidal, anti-leishmanial, cytotoxicity, antibacterial and antifungal activities. Sample E2 appeared to have significant phytotoxic activity. In the insecticidal screening study only one of the insects, *Rhyzopertha dominica, *showed acceptable mortality after treatment with E2, but weak response to E3 and E4 fractions. Leishmanicidal activity of the chloroform fraction was present, but not very significant. E2 showed favorable cytotoxic activity, while E3 had weak activity, and the other samples had no significant activity. In the antifungal screening tests; E2, E3 and E4 fractions exhibited about 25% inhibition of fungal growth against *Fusariumsolani*, while they were not active against other tested fungi. The results of antibacterial screening were completely negative, which may be due to resistance towards these types of constituents.

## Introduction

Euphorbiaceae is one of the largest families of the phylum Anthophyta. In this family, the largest genus is *Euphorbia*, which comprises well over 2000 species, grows in the form of laticiferous herbs, shrubs, and small trees, inhabiting the tropical and temperate zones of Asia and other parts of the world (1). For centuries, plants and plant materials of *Euphorbia *have been known to be poisonous to human beings. Often they are held responsible for the poisoning of livestock and are used as arrow poisons. In traditional medicine, it was used as treatment of intestinal parasites, gonorrhea and in treatment of skin diseases (2, 3). However, multidisciplinary pharmacological screening carried out on a variety of ailments like cancer, rheumatism, neuralgia, asthma, and bacterial infections (2) and its latex is one the remedies used topically to treat the coetaneous leishmaniasis (4, 5). Therefore, secondary bioactive metabolites of this plant seem to be active against a wide range of assay targets. Thus, plant extracts suggested to screened for general biologic activities, throw out the negatives and then run specific bioassays on actives, the active fractions selected, and the bioactive compounds isolated and identified. 

In this research *Euphorbia Aellenii, *a perennial plant growing in some parts of Iran (6), was selected to perform bioassay tests as a guide for selection of bioactive fractions to avoid the risk of wasting time and purification of undesired compounds. The following six bioassays:

phytotoxicity, insecticidal antileishmanial, cytotoxicity, antibacterial and antifungal activity, focused on pharmacological and folk medicine rationales, as inexpensive, rapid, and simple top-benched general bioassays were applied as a strategy for screening, fractionation and monitoring of physiologically active natural products in this plant.

## Experimental

*Plant material*

The whole plant of *Euphorbia aellenii *(Euphorbiaceae) was collected in August 2007 from populations growing in Galil-e-Shirvan (near the Turkmenistan border), Northern Khorasan province, Iran. The plant was identified by Mrs. Yasamin Naseh, plant taxonomist (Department of Botany, Herbaceous Sciences Research Center, Ferdowsi University, Mashhad, Iran). A voucher specimen of the plant was deposited in the herbarium of the Pharmacognosy Department, Faculty of Pharmacy and Pharmaceutical Sciences, Isfahan University of Medical Sciences, Iran.

*Extraction and isolation*

The dried plant (7 Kg) was extracted three times with MeOH (20 L) at room temperature for 4 days and the resulting extract was then concentrated to a dark green gum. The gummy residue (500 g) partitioned between *n*-hexane and MeOH. The defatted MeOH extract was evaporated and dissolved in water to make a suspension and then partitioned with different solvents including chloroform, ethyl acetate and *n*-butanol, respectively. Four fractions, which were the chloroform (E2), ethyl acetate (E3), *n*-butanolic (E4) and aqueous (E5) samples were obtained and subsequently investigated for different biological activities.

*Phytotoxic activity*

In order to conduct the *Lemna minor *method for phytotoxicity assay, an inorganic medium was prepared by mixing various inorganic constituents in 1 L distilled water, adjustment of pH between 5.5 to 6.0 using KOH, and autoclaving the medium at 121oC for 15 min. 

Next, weighed 15 mg samples of E2, E3, E4, E5, as well as paraquat (*N*, *N*′-dimethyl-4, 4′- bipyridinium dichloride) as the standard herbicide agent were individually dissolved in 15 mL of ethanol, and used as stock solutions. Then 1000 mL , 100 mL and 10 mL increments of these stock solutions were added into different vials and after overnight evaporation of the solvent, 2mL of the prepared medium was added into each vial to make concentrations of 500, 50 and 5 ppm, respectively. After that a single fresh, green plant containing a rosette of three fronds was added to every vial. They were then individually placed in a Petri dish filled with about 2 cm of water, and sealed with greasy glass plate. Petri dishes were then placed in the growth chamber for one week at 28 ± 1°C, under a fluorescent lamp. The number of fronds per vial were counted and recorded on the seventh day (7). The results were calculated using the following equation and with reference to paraquat as the standard herbicidal agent, as well as the volatile solvent as the negative control (7): 

% Regulation = 100 – Number of fronds in the test sample × 100 Number of fronds in – the negative control 

The criteria used were as follows: 0-39% inhibition (low activity), 40-59% inhibition (moderate activity), 60-69% inhibition (good activity), above 70% inhibition (significant activity). 

*Insecticidal activity*

Samples E2, E3, E4 and E5 were evaluated against different insects. At first, samples were prepared by dissolving 200 mg of each sample, individually, in 3 mL ethanol. Next, filter papers impregnated with these sample solutions, a concentration of 1 mg/cm^2^, were prepared and left intact for 24 h for solvent evaporation. From every insect, ten adult insects were transferred to Petri dishes covered by the impregnated filter papers. This procedure was also performed for the negative control (without any sample) and the positive control (using permethrin as the standard agent at a concentration of 239.5 μg/cm^2^ of filter paper). The survival of insects after 24 h of direct contact with the filer paper impregnated with the test sample was assessed (8). 

The results were calculated using the following equation, as the percentage of mortality, with reference to permethrin, as the standard drug at a concentration of 239.5 μg/cm^2^ as the positive control and volatile solvent as the negative control (7-8). 

% Mortality = [1- (Number of insects alive in test/ Number of insects alive in –the negative control)] × 100 

*Anti-leishmanial activity bioassay*

Leishmanialpromastigotes were cultured in a sterile 25 cm2 tissue culture flask containing buffered M-199 medium along with 25 mM HEPES (7) and 10% heat inactivated foetal bovine serum at pH 7.2 at 25 °C. Parasites were centrifuged at 3000 rpm, diluted with PBS and counted using a Neubauer chamber viewed under an optical microscope. Then, parasites were diluted with fresh medium to a concentration of 2×10^6^ /mL. Stock samples were prepared by dissolving 1 mg in 50 μL DMSO and making the volume up to 1 mL with the culture medium. In the wells of a 96 well micro-titer plate, 90 μL of the parasite culture (2×10^6^ /mL) along with 10 μL of different concentrations of stock samples (serial two fold dilutions) were added. 

Ten μL of PBS (phosphate buffered saline, pH 7.2 containing 0.5% DMSO) was added as the negative control, while amphotricin B and pentamidine at a concentration of 1 mg/mL were added separately as the positive controls. The plate was incubated at 22 ^o^C for 72 h, and the amount of parasites in each well was determined microscopically, using a Neubar chamber (7, 9).

*Brine shrimp (Artemiasalina) cytotoxicity*

For the brine-shrimp lethality assay, at first the artificial sea-water was prepared by adding sea-salt at a concentration of 3.8 g/L to double distilled water, followed by filtration of the resulting solution. Then the sea-water was poured into an aluminum foil covered tank, and 1 mg of shrimp eggs was added to this tank. Brine shrimp (*Artemiasalina *Leach) nauplii were hatched and matured after two days. Next, we selected some vials containing 1000, 100 and 10 μg/mL concentrations were prepared. After that stock sample solutions were prepared by dissolving 20 mg/2mL of samples in the volatile solvent, followed by the addition of 500, 50 and 5 μL of these stock solutions to different vials sequentially. After evaporation of the solvent, dried samples were again dissolved in 50 μL DMSO and 5 mL sea-water was added to make concentrations of 1000, 100 and 10 μg/mL, respectively. Ten shrimps were transferred to each sample vial and kept under illumination for 24 h. Finally, surviving shrimps were counted and data recorded were used to obtain LC_50 _and 95% confidence intervals (7).

*Antifungal activity*

For the evaluation of antifungal activities of test samples, they were tested against *Candida albicans*, *Aspergillusflavus*, *Microsporumcanis*, *Fusariumsolani*, and *Candida glaberata*, using the tube dilution protocol as the preliminary antifungal screening test. For preparation of test samples, 24 mg of samples were dissolved in 1 mL of sterile DMSO, serving as stock solutions. Sabouraud dextrose agar (SDA) was used for the growth of fungus. The acidic (pH value of 5.5 to 5.6) medium containing a relatively high concentration of glucose or maltose 2 was prepared, to give a concentration of 32.5 g/500 mL in distilled water. It was then steamed to dissolve the contents and dispensed in a known amount into screw cap tubes, followed by autoclaving at 121°C for 15 min. For loading the samples, tubes were allowed to cool to 50°C and non-solidified SDA was loaded with samples from the stock solution to make a final concentration of 400 μg/mL. Tubes were then allowed to solidify in a slanting position at room temperature. Each tube was inoculated with a 4 mm diameter piece of inoculum, removed from a seven day old fungal culture. For the non-mycelial growth, an agar surface streak was employed. Other media supplemented with DMSO and reference antifungal drugs were also prepared and used as the negative and positive controls, respectively. The tubes were incubated at 27-29°C for 7 days. Cultures were examined twice weekly during the incubation period. Evaluation of growth within the amended media was determined by measuring the linear growth (mm) and growth inhibition, calculated with reference to the negative control using the following formula:

% Inhibition of fungal growth = 100 – linear growth in test sample (mm) × 100 linear growth in control (mm)

The results were categorized as low (0-39%), moderate (40-59%), (60-69%) and significant (above 70%) activity, respectively.

The standard drugs used in the assays were miconazole and amphotericin B (7, 10).

*Antibacterial bioassay*

The agar well diffusion method was used as the preliminary screening test of *in vitro *antibacterial bioassay. In the first day a single colony of bacterial culture in nutrient broth was inoculated and incubated at 37°C for 24 h. Then, in the second day a soft agar tube was taken, melted and cooled up to 45°C, followed by the addition of 10 μL of fresh bacterial culture. After shaking and pouring it on to the nutrient agar containing plate, the plate was rotated to make even distribution of the culture and allowed to solidify. Wells were made by using 6 mm-diameter sterile borer and labeled with the sample code. Stock solutions of test samples (E2- E5) were prepared at a concentration of 3 mg/mL in DMSO (as solvent) and 100 μL of dilutions were poured into respective wells and other wells supplemented with DMSO and reference antibacterial drug (imipenem) at a concentration of 10 μg/mL/disc, serving as the negative and positive controls, and incubated at 37°C for 24 h. In the next day, results were noted in terms of the zone of inhibition in mm and interpreted, based on the following criteria: 0 = no activity, 9-11 mm = not significant, 12-14 mm = low activity, 15-17 mm = good activity, and above 18 mm = significant activity

Presence of antibacterial agent was indicated by the growth inhibition of the bacterial strains and appearance of zone of inhibition (observation of clear zone where the growth of bacteria had not occurred) (7).

## Results

*Phytotoxicity assay*

Bioactivity of all the extracts (E2-E5) was investigated under the incubation condition of 28 ± 1°C and at three different concentrations of 1000, 100 and 10 μg/mL. Sample E2 appeared to have good phytotoxic activity, with 65% inhibition of the *Lemna minor *growth at a high concentration (1000 μg/mL) and moderate weedicidal activity at 100 μg/mL concentration. Other extracts (E3, E4 and E5) showed a moderate response (40-45%) at a concentration of 1000 μg/mL. All the samples were weakly active (5-10%) at a concentration of 10 μg/mL . The results of phytotoxic activity of the test compounds have been shown in Table 1. 

**Table 1:** *In vitro *phytotoxic bioassay of different fractions of *Euphorbia Aellennii*a

Samples^b^	**Concentration (μg/mL)**	**Number of fronds**		**%growth regulation**	**Concentration of the standard drug (μg/mL)**
		**Sample**	**Control**		
E2	1000	9	20	65	0.015
E3		12		40	
E4		11		45	
E5		12		40	
E2	100	15	20	35	0.015
E3		15		25	
E4		14		30	
E5		16		20	
E2	10	18	20	15	0.015
E3		19		5	
E4		19		5	
E5		19		5	

^a^Standard drug: Paraquat; number of replicates = 3; Incubation condition = 28 ± 1°C

^b^Chloroform (E2), ethyl acetate (E3), *n*-butanolic (E4) and aqueous (E5) fractions.

*Contact insecticidal toxicity*

Three adult insects (*Tribolium castaneum, Rhyzopartha dominica and Callosobruchus analis*) were used for the direct contact insecticidal bioassay. Only one of the insects, *Rhyzopertha dominica*, showed acceptable mortality after treatment. E2 caused 45.56% mortality, determined as moderate activity. E3 and E4 showed 20% mortality, determined mortality. No activity was found with E5, against *Rhyzopertha dominica*. Other insects were not found to be susceptible to the tested compounds, as shown in Table 2.

**Table 2:** Insecticidal activity obtained by the contact toxicity method on different fractions of *Euphorbia Aellennii*^a^

**Name of Insect **	**% Mortality **					
	**Positive control **	**Negative control **	**E2 ** ^b^	**E3 **	**E4 **	**E5 **
*Triboliumcastaneum*	100	0	0	0	0	0
*Rhyzoperthadominica*	100	0	45	20	20	0
*Callosbruchusanalis*	100	0	0	0	0	0

^a^Standard drug: Premethrine; concentraion of sample = 1019 μg/cm^2^ and premethrine = 239 μg/cm^2^; number of replicates = 3.

^b^Chloroform (E2), ethyl acetate (E3), *n*-butanolic (E4) and aqueous (E5) fractions.

*Anti-leishmanial activity bioassay*

In this bioassay method all assays were run in duplicate and amphotricin B and pentamidine (both at a concentration of 1 mg/mL), as well as the chloroform fraction of *E. Aellenii *(1 mg/mL) in DMSO, showed a growth inhibition effect on the *Leishmania major *promastigotes in a dose-dependent manner, while solvent did not have any effect. The IC_50_ value, indicating the effective concentration of compound in μg/mL necessary to achieve 50% growth inhibition, was found to be 140 ± 24 μg/mL. The value obtained for amphotricin B and pentamidine were 0.29 ± 0.05 μg/mL and 5.09 ± 0.04 μg/mL, respectively. While other samples (E3-E5) showed no significant activity.

*Brine shrimp (Artemiasalina) cytotoxicity*

LD_50_ measurements of E2-E5 fractions were investigated against *Artemiasalina *brine-shrimp eggs. Fraction E2, showed cytotoxicity at a concentration of 1000 μg/mL . E3 exhibited weak lethality, with a LD_50 _value of 770.66 μg/mL. Its lowest toxic concentration was found to be 74.07 μg /mL. Other test samples (E4 and E5), as shown in Table 4, found to be non-cytotoxic and had no significant lethality on brine shrimps. The results of cytotoxicity activity of the compounds are shown in Table 3.

**Table 3:** Brine shrimp (*Artemiasalina*) lethality bioassay of different partitions of *Euphorbia Aellenii*

**Sample** ^a^	**Dose** **(μg/mL)**	**Number of** **shrimps**	**Number of** **survivors**	**LD**_50_ **(μg/mL)**	**Standard drug**	**LD**_50_ **(μg/mL)**
E2b	1000	30	10	177.06	Etoposide	7.4625
	100	30	18			
	10	30	21			
E3	1000	30	19	770.66	Etoposide	7.4625
	100	30	23			
	10	30	26			
E4	1000	30	26	5346200	Etoposide	7.4625
	100	30	27			
	10	30	28			
E5	1000	30	24	377166.8	Etoposide	7.4625
	100	30	26			
	10	30	28			

^a^Chloroform (E2), ethyl acetate (E3), *n*-butanolic (E4) and aqueous (E5) fractions.

^b^G (Probability value) = 0.48; No. of replicates: 3; Upper limit (upper toxic concentration) = 1878.68 μg/mL and lower limit (lower toxic concentration) = 42.38 μg/mL; incubation condition = 28 ± 1°C

*Antifungal bioassay*

E2, E3 and E4 fractions exhibited weak antifungal activities against *Fusarium solani*, with a fungal growth inhibition of about 25-26% (Table 5). Whereas , the result of aqueous fraction (E5) was not significant. None of the tested compounds were found to be active against *Candida albicans*, *Aspergillus flavus, Microsporum canis*and *Candida glabrata*, when using a concentration of 400 μg/mL of the test samples (Table 4). 

**Table 4:** *In vitro *antifungal bioassay (agar tube dilution protocol) results

Name of fungus	Linear growth (mm)					% Inhibition					MIC of the standard drug (μg/mL)
	E2a	E3	E4	E5	Negative control	E2	E3	E4	E5	Negative control	
***Candida albicans***	100	100	100	100	100	0	0	0	0	0	Miconazole (108.00)
***Aspergillusflavus***	100	100	100	100	100	0	0	0	0	0	Amphotericine B (20.02)
***Microsporumcanis***	100	100	100	100	100	0	0	0	0	0	Miconazole (98.40)
***Fusariumsolani***	75	74	74	100	100	25	26	26	0	0	Miconazole (73.25)

^a^Chloroform (E2), ethyl acetate (E3), *n*-butanolic (E4) and aqueous (E5) ftions

^b^Concentration extraction of samples = 400 μg/mL of DMSO; replicates = 3; incubation period = 7 days; incubation temperature = 28 ± 1°C.

*Antibacterial bioassay*

The antibacterial study was performed against *Escherichia coli, Bacillus subtilis, Shigella flexenari, Staphylococcus aureus, Pseudomonas aeruginosa *and *Salmonella typhi. *The data obtained, showed that samples E2, E3, E4 and E5 were totally ineffective.

## Discussion

Chloroform fraction of the methanolic extract of *Euphorbia Aellenii*appeared to have good phytotoxic activity at a concentration of 1mg/mL and moderate weedicidal activity at a concentration of 100 μg/mL . The other fractions (ethyl acetate, butanolic and aqueous) showed moderate response (40-45%) at a concentration of 1000 μg/mL and low activity in lower concentrations. In the case of *Euphorbia wallichi*, as reported by Irshad Ali and co-workers, chloroform and ethyl acetate fractions showed significant activity at a concentration of 100 μg/mL (11). However, in a research conducted on *Euphorbia helioscopia*by Uzair *et al., *the crude methanolic extract had non-significant phytotoxicity against *Lemna minor *(12). These data would suggest the potential of the chloroform fraction for further phytochemical analysis, as a potent herbicide.

The chloroform fraction of *Euphorbia Aellenii*(1 mg/mL) in DMSO, showed agrowth inhibitory effect on *Leishmania major *promastigotes, with an IC_50_ value of 140 ± 24 μg/mL compared with amphotricin B (0.29 ± 0.05 μg/mL) and pentamidine (5.09 ± 0.04 μg/mL). In fact, samples with IC_50_ values greater than 25 μM are considered to be weakly active. However, based on the results obtained from the extract, it is proposed that the active leishmanicidal components may be present in very low amounts, and suggesting the need for purification of secondary metabolites in order to achieve a more favorable leishmanicidal activity (7). In a study conducted by Ja’fari and co-workers at 2005 on *Euphorbia myrsinites*, the methanolic extract at a concentration of 1 mg/mL had favorable leishmanicidal activity and killed the *Leishmania major *promastigotes in a dose-dependent manner, with an EC50 value between 0.5 and 0.25 mg/mL (9). In another research on *Euphorbia lagascae*(13), stilbenes possessed moderate anti-leishmanial activity against promastigotes and also taxane type diterpenoids, which are structurally similar to myrisinane type diterpenoids, present in the *Euphorbia *genera had shown anti-leishmanial activity (14). Some cycloartane type triterpenes have also shown anti-leishmanial activity (15) and due to the presence of phenolic compounds like stilbenes, myrisinane type diterpenoids and cyloartanes in the *Euphorbia *genus, the anti-leishmanial activity could be related to these types of compounds.

In the insecticidal bioassay, the chloroform fraction caused moderate activity and ethyl acetate and butanolic fractions had weak mortality against only one of the insects, *Rhyzopertha dominica*. In a study by Civelek and Weintaub, *Euphorbia myrsinites *showed acceptable insecticidal activity (16). Furthermore, in traditional medicine *Euphorbia *has been used in the treatment of intestinal parasites and as pesticide (2). These data candidate the chloroform fraction for further phytochemical analysis, in order to discover new compounds with insecticidal activity.

This study also showed in the cytotoxicity activity of the chloroform fraction of *Euphorbia aellenii*, which was comparable with the chloroform fraction of *Euphorbia wallichi *(11). This rather good cytotoxicity effect was seen at a high concentration of 1000 μg/mL, in compliance with many other literature reports on the genus *Euphorbia *for cytotoxic effect due to the presence of diterpenoid polyesters. Based on this study, it is recommended to purify and isolates the diterpenoid polyesters, as a good candidate for anticancer activity, and compare it with taxol and deacetyl-baccatine as known diterpenoid anticancer drugs, and other diterpenoids found in Euphorbiaceae.

In terms of the antifungal screening test, all the fractions exhibited rather low percentage of fungal growth inhibition against *Fusariumsolani*. However, in a similar work on *Euphorbia wallichii, *no significant antifungal activity was observed for the tested fungi (11). 

The result of antibacterial screening was totally negative while antibacterial study performed against bacteria on ethyl acetate partition of *E. wallichi*(11) and ethanolic extract of *E. peplus*(17) have showed antibacterial activity. In traditional medicine also there are some reports of using euphorbia against some bacterial infections like gonorrhea (2) thus, the negative result may be due to a resistance to this type of constituents.
